# Notes and News

**Published:** 1905-09-09

**Authors:** 


### Notes and News.

The King has sent his annual subscription of one hundred guineas to King Edward's Hospital Fund.

The balconies at the North-Eastern Hospital for Children have proved so beneficial to the small patients that the authorities intend to add balconies to the old wards when money is forthcoming. £8,700 is, however, still owing to pay off the mortgage on the existing balconies.

The Inter-Continental Railway Company promises to do a good service by the establishment of a special fruit service from the Continent, whereby the more delicate kinds of fruit can reach their destination after a very long journey comparatively uninjured. To secure this the fruit travels in cold chambers direct to its destination, which is quite a new departure in transporting.

Sir John Gorst delivered a very emphatic address before a mass meeting of gasworkers and labourers at Hanley this week. His subject, to which he has devoted so much attention, was "Infantile Rearing and Mortality, and the Care of the Sick by the State." In the latter part of his address he made allusion to the Municipal Hospitals of Germany, to which everyone had a right to attend, and those who were able were expected to pay according to their means. Sir John Gorst compared our system unfavourably with the German method, as affording less privilege to the poor. But his audience must have been well aware that there is no lack of free hospital relief in England, and that the payment according to means system would be far from popular if introduced amongst them.

Plague is reported again in Zanzibar and at Mombasa.

Last week we announced the fact that the removal of the Hastings Hospital was contemplated. We now learn that the authorities have received an offer of £20,000 for the site, upon which it is proposed to erect a kursaal. A meeting of Governors was held at which Lord Brassey took the chair. Lord Brassey pointed out that if the Governors did not accept the offer they would have to spend money in adding to the hospital. A new operating-room and a house for the nurses' home was required. It was pointed out that a site of about three acres was available at a cost of £3,000. £2,000 was estimated as the worth of the building materials of the hospital, and the borough engineer expressed a belief that it was quite possible to provide a new hospital of 100 beds for £18,000. The medical staff appear to be unanimous in desiring removal from a site regarded as unsuitable and noisy.

Professor Boyce, of the Liverpool University, is to visit Belize, in British Honduras, to report on the sanitary measures in that colony necessary in view of the recent outbreak of yellow fever. Professor Boyce is now at New Orleans, completing his observations of the methods employed by the Americans in combating yellow fever there. Both members of the yellow fever expedition of the Liverpool School at Manaos, in Brazil, have been ill with yellow fever, one very seriously. The latter has now been invalided to Madeira to recuperate, but proposes to return to continue his work. The expedition express the hope that they will now be immune. The medical officers of Manaos have shown them the greatest attention and kindness during their illness. The surviving members of the sleeping sickness expedition which the school sent to the Congo in August 1903 are now returning to England.

Mr. E. G. Bawden's munificent gift of £100,000 to charities has been allocated with Mr. Edgar Speyer's assistance. We are glad to see that £16,000 is given to the New University of London Scheme. King Edward's Hospital Fund is to receive £5,000, whereby all the hospitals in the metropolis will benefit. The London Hospital also receives £5,000, the City of London Hospital for Diseases of the Chest, the Poplar Hospital for Accidents, and the National Hospital for the Paralysed and Epileptic each receive £3,000, and £2,500 each is to be given to the Royal Waterloo Hospital for Children and Women, the Cancer Hospital, and the German Hospital. Queen Charlotte's and the Royal Hospital for Incurables each receive £2,000, and the Clapham Maternity Hospital £1,000. Five convalescent homes, and numerous homes and institutions each receive either £2,000 or £1,000. We are sorry to see that the Metropolitan Hospital and the West Ham Hospital which do such an excellent, and much needed work in Mr. Bawden's neighbourhood, have not been selected for a special gift. They have few rich neighbours to help, and consequently their existence is apt to be overlooked by those upon whom they depend for support.

